# Icariin Mitigates Cognitive Impairments in Female Rats with Estrogen Deficiency

**DOI:** 10.1002/alz.087209

**Published:** 2025-01-09

**Authors:** Ahsas Goyal, Vibhav Varshney, Kritika Aggarwal

**Affiliations:** ^1^ Institute of Pharmaceutical Research, GLA University, Mathura, Uttar Pradesh India; ^2^ B.S.A. College of Engineering and Technology, Mathura, Uttar Pradesh India

## Abstract

**Background:**

Cognitive dysfunction emerges as a manifestation of reduced estrogen levels following ovariectomy in an individual. However, the conventional use of estrogen replacement therapy could increase the risk of breast cancer and thromboembolism. Icariin is a natural compound that has been reported to be a neuroprotective agent against dementia. However, the therapeutic role of the icariin via Nrf‐2‐mediated neuroprotective effect to improve learning and memory is yet to be evaluated in ovariectomized challenged female rats.

**Method:**

Experimentally, dementia was induced by the bilateral ovariectomy of female Wistar rats on the first day (Day‐1) of the experimental protocol of 60 days. Biochemical estimation was done on last day of the protocol to evaluate the cholinergic function using commercially available kits.

**Result:**

Intraperitoneal administration of icariin (Day‐1 to Day‐60) significantly improved the learning and memory activity during the MWM schedule and increased the spontaneous alternation behavior during the performance of the Y maze. Further, icariin increased the activity of ChAT and the level of ACh respectively, and decreased the activity of AChE in HIP, PFC, and AMY. Additionally, the improved level of LPO, SOD and CAT activity have been observed in HIP, PFC and AMY brain regions after the administration of icariin. Moreover, Nrf‐2 antagonist significantly attenuated the therapeutic effect of icariin in ovariectomized‐induced altered behavioral and neurochemical observations in female rodents.

**Conclusion:**

Thus, these above facts suggested that icariin exerts the Nrf‐2‐mediated neuroprotection in ovariectomized rats indicating that icariin also should be taken into consideration as a therapeutic option in the management of estrogen deficit‐induced cognitive dysfunctions.

